# Examining the relationship between early childhood temperament, trauma, and post-traumatic stress disorder

**DOI:** 10.1016/j.jpsychires.2021.10.004

**Published:** 2021-12

**Authors:** Chantelle Wiseman, Jazz Croft, Stan Zammit

**Affiliations:** a Population Health Sciences, School of Medicine, University of Bristol, United Kingdom; b Division of Psychological Medicine and Clinical Neurosciences, School of Medicine, Cardiff University, United Kingdom

**Keywords:** PTSD, Temperament, Trauma, Cohort study, ALSPAC

## Abstract

A greater understanding of why some people are more at risk of developing PTSD is required. We examine the relationship between temperament traits in early childhood and subsequent trauma exposure and risk of PTSD. We used data on 2017 participants from the Avon Longitudinal Study of Parents and Children (ALSPAC). Temperament was measured using the Carey Infant Temperament Scale (average score from ages 6 and 24 months). This provided data on 9 individuals traits, and Easy, Medium, and Difficult temperament clusters. Trauma exposure was measured from 0 to 17 years, and PTSD at age 23 years using the PTSD Checklist for DSM-V (PCL-5). Regression models were used to estimate associations between temperament and both trauma and PTSD, and to examine mediation (of temperament to PTSD pathway) and interaction (temperament X trauma on PTSD) effects. 1178 (58.4%) individuals were exposed to a trauma in childhood and 112 (5.5%) had PTSD. Higher levels of Intensity were associated with a small increase in trauma exposure (OR_adjusted_ 1.23, 95% CI 1.12, 1.34; *p* < 0.001) and PTSD (OR_adjusted_ 1.27, 95% CI 1.05, 1.54; *p* = 0.012). Higher levels of Activity, Adaptability, Mood and Threshold temperament traits were also associated with trauma exposure. Medium (OR_adjusted_ 1.49, 95% CI 1.21, 1.84; *p* < 0.001) and Difficult (OR_adjusted_ 1.47, 95% CI 1.18, 1.84; *p* = 0.001) temperament clusters were associated with increased trauma exposure compared to an Easy cluster, but were not associated with PTSD. The relationship between trait Intensity and adult PTSD was partially mediated by childhood/adolescent trauma (Indirect OR_adjusted_ 1.08, 95% CI 1.01, 1.16, *p* = 0.024, proportion mediated 26.2%). There was some evidence that trait Intensity modified the relationship between trauma and PTSD (OR_adjusted_ 1.66, 95% CI 1.07, 2.55, *p =* 0.023). PTSD in early adulthood is more common in those with intense stimuli responsiveness in childhood. Temperament traits might be useful predictors of trauma exposure and mental health outcomes and offer potential targets for supportive interventions.

## 1 Introduction

Post-traumatic stress disorder (PTSD) can occur following exposure to a traumatic event such as a near-death experience or a physical or sexual assault (American Psychiatric Association, 2013). It is characterised by three key symptom clusters: re-experiencing of the event, avoidance of reminders of the trauma, and an exaggerated threat response (World Health Organization, 2019). More recent classification systems also recognise complex PTSD, usually occurring following repeated exposure to trauma, often in childhood, and characterised by an additional symptom cluster of disturbances in self-organisation (World Health Organization, 2019). The lifetime risk of PTSD is approximately 8% (Kessler et al., 1995) and it is a common and frequently undetected co-morbid disorder in psychiatric secondary care services (Zammit et al., 2018). A greater understanding of who is most at risk of being exposed to trauma, and of developing PTSD after such exposure, is required to inform efforts to reduce the public health burden associated with PTSD.

Trauma is a necessary, but not sufficient, cause for the development of PTSD, i.e. not everyone who experiences trauma will develop PTSD. Although factors directly relating to the trauma, such as type of trauma (Bryant, 2019) and level of threat to life (Trickey et al., 2012; Ozer et al., 2003), show the strongest association with risk of developing PTSD, other pre- and peri-trauma characteristics are also associated with higher PTSD risk, including level of social support around the time of the trauma (Trickey et al., 2012; Ozer et al., 2003), pre-existing mental illness (Brewin et al., 2000), family history of mental disorder (Ozer et al., 2003; Brewin et al., 2000; Breslau, 2002), lower cognitive ability (DiGangi et al., 2013) and female sex (Breslau, 2002).

Both cross-sectional and longitudinal studies repeatedly show a relationship between personality and PTSD, particularly neurotic personality traits (Breslau, 2002; Cox et al., 2004; Mattson et al., 2018) but also avoidant traits and hostility (Breslau, 2002). Additional individual characteristics such as lower self-directedness and low cooperativeness (North et al., 2012), high harm avoidance (North et al., 2012; Kampman et al., 2017), lower hardiness (Krauss et al., 2018) and negative affect and appraisal (DiGangi et al., 2013) are also associated with PTSD. Whilst these findings suggest that personality characteristics can influence the development of PTSD, teasing out whether personality characteristics in adulthood preceded or occurred following onset of PTSD in many of these studies was not possible.

One way to help establish whether relatively stable individual differences in emotions and behaviour (that are captured by personality trait measures) affect risk of developing PTSD is to examine temperament, which can be measured in early childhood, before exposure to (most) trauma or development of PTSD occurs. Temperament describes the affective and behavioural characteristics of children, is relatively stable, and correlates moderately with adult personality (Rothbart et al., 2000). One of the most established measures of temperament derived from the New York Longitudinal Study, which assesses nine different traits (Thomas et al., 1970), which can also be used to derive three overarching clusters (Easy, Slow to Warm and Difficult) that describe the overall behaviour of the child. A difficult temperament describes children who are characterised by higher negative mood, withdrawal, and intensity, and lower adaptability and regularity (Thomas et al., 1970) and is associated with the most adverse outcomes (Bates et al., 1991, cited in Micalizzi et al., 2017).

Individual temperament traits, most notably intensity of reaction and emotionality have also been associated with various psychiatric disorders in children and adolescents (Sayal et al., 2014; Bould et al., 2014).

A difficult temperament in childhood has been associated with PTSD in the Dunedin birth cohort (Koenen et al., 2007), but as far as we are aware no other longitudinal studies have examined the relationship between childhood temperament and PTSD. Children with more difficult temperaments are more likely to elicit negative parenting (Micalizzi et al., 2017), and to develop behavioural problems (Bates et al., 1991, cited in Micalizzi et al., 2017), including drug use, and therefore associations with PTSD might be a result of greater exposure to traumas. However, temperament traits have also been associated with different reactions to trauma; for example, high levels of negative emotionality has been associated with increased risk of developing psychiatric symptoms after interpersonal violence (Koenen et al., 2007; Yalch et al., 2017), and thus temperament might modify the effects of trauma on PTSD risk. Establishing whether and how temperament traits are associated with risk of developing PTSD could help inform understanding of aetiology and prediction of this disorder, and help establish whether the personality traits and characteristics identified in individuals with PTSD precede the disorder or are symptomatic of it. The aim of this study is to examine, using a cohort study design, whether individual temperament traits in early childhood are associated with trauma exposure through childhood and adolescence, and with PTSD in early adulthood. We also examine whether the relationship between temperament and PTSD is mediated by trauma, and whether the association between trauma and PTSD is modified by temperament.

## 2 Materials and methods

### 2.1 Study population

This study examined participants from the Avon Longitudinal Study of Parents and Children (ALSPAC) birth cohort. Pregnant women resident in Avon, UK with expected dates of delivery April 1, 1991 to December 31, 1992 were invited to take part in the study. The initial number of pregnancies enrolled was 14,541, and of these initial pregnancies, there was a total of 14,676 foetuses, resulting in 14,062 live births and 13,988 children who were alive at 1 year of age. ALSPAC provides a wealth of data collected via questionnaires, clinic appointments and interviews on potential determinants of health (Boyd et al., 2013; Fraser et al., 2013; Northstone et al., 2019). Please note that the study website contains details of all the data that is available through a fully searchable data dictionary and variable search tool: http://www.bristol.ac.uk/alspac/researchers/our-data/

For this study we examined 2017 participants who had data on childhood temperament measures at 6 and 24 months, measures of trauma between ages 0 and 17 years, PTSD at age 23 years, as well as information on confounders (see Suppl. Fig. 1 for flow chart).

Ethical approval for the study was obtained from the ALSPAC Ethics and Law Committee and the Local Research Ethics Committees. Anonymised data is used for this study. Data were analysed between March and June 2020.

### 2.2 Measures

#### 2.2.1 Exposure

##### 2.2.1.1 Temperament traits

Temperament was assessed at 6 months and 24 months of age using the parent-reported Carey Infant/Toddler Temperament Scale (CITTS) questionnaire (Carey, 1970). The CITTS is a validated tool that measures nine temperament traits: *Activity* (degree of movement of the child), *Rhythmicity* (predictability of biological functions such as sleeping), *Approach* (how well the child responds initially to a new stimulus), *Adaptability* (later response to this change in environment), *Intensity (of reaction* - strength of response to a stimulus, regardless of whether this is positive or negative), *Threshold (of responsiveness-*level of physical stimulus needed to provoke a reaction), (*Quality of) Mood* (whether often happy or sad), *Distractibility* (whether responds easily to diversions or remains focussed), and *Persistence* (length of time can focus on one task). The CITTS is a parent-reported questionnaire and higher scores on each trait are indicative of a more difficult temperament (Carey, 1970).

There was weak to moderate intra-trait stability over time (6 and 24 months), apart from Distractibility (r = −0.05; Suppl. Table 1). We created a mean score for each trait across the two time points, and these were then standardised for ease of interpretation. All trait measures were normally distributed. Cronbach's alpha was 0.72 for these nine temperament traits, and Table 1 shows the correlations between them.

**Table 1:** Pearson's correlation between different temperament traits.

	Activity	Rhythmicity	Approach	Adaptability	Intensity	Mood	Persistence	Distractibility	Threshold
**Activity**	1.00	–	–	–	–	–	–	–	–
**Rhythmicity**	0.05	1.00	–	–	–	–	–	–	–
**Approach**	−0.04	0.18	1.00	–	–	–	–	–	–
**Adaptability**	0.23	0.31	0.50	1.00	–	–	–	–	–
**Intensity**	0.46	0.09	0.09	0.30	1.00	–	–	–	–
**Mood**	0.18	0.33	0.53	0.64	0.23	1.00	–	–	–
**Persistence**	0.09	0.24	0.21	0.34	−0.03	0.41	1.00	–	–
**Distractibility**	0.09	0.21	0.31	0.42	0.10	0.41	0.34	1.00	–
**Threshold**	0.16	0.00	0.17	0.13	0.35	0.07	−0.08	0.16	1.00

##### 2.2.1.2 Temperament clusters

We also simplified these nine temperament traits into three temperament clusters, Easy, Medium and Difficult by summing the scores of the five most important temperament traits that contribute to a difficult temperament (Rhythmicity, Approach, Adaptability, Intensity and Mood) (Carey, 1970) and dividing this summed score into tertiles.

#### 2.2.2 Outcomes

##### 2.2.2.1 Trauma

A binary measure of trauma was derived from 121 questions selected from 48 assessments completed contemporaneously by the child or their parents from the ages of 0–17 years, and from a questionnaire completed by the young person at age 22 to supplement information on sexual abuse that was almost entirely parent-reported in earlier questionnaires. The questions covered sexual, physical and emotional abuse, as well as emotional neglect, bullying and exposure to domestic violence, and were selected on the basis that they would be deemed as being highly upsetting for almost everyone who experienced them. Participants were coded as having been exposed to a trauma if they endorsed any of the questions relating to these traumas between ages 0–17 years. Participants were coded as non-exposed if they had not endorsed any of the questions, and participated in at least 50% of assessments. For further details on how this measure was derived see Croft et al. (2018). This method allowed us to establish a comprehensive measure of trauma exposure during the developmental period, measured at multiple time-points using a variety of assessments.

##### 2.2.2.2 PTSD

At age 23, 4087 participants completed a modified version of the Life Events Checklist that asked about directly-experienced events (Weathers et al., 2013a), and those who endorsed any lifetime trauma on this (N = 1813) also completed the PTSD Checklist for DSM 5 (PCL-5) (Weathers et al., 2013b). This consists of 20 items covering the core symptoms of PTSD. Each item is scored from 0 to 4, and items can be summed to provide a total score (0–80). Cronbach's alpha was 0.96. We looked at two outcome measures of PTSD. Firstly, a binary variable of a probable DSM-V diagnosis of PTSD, defined as the presence of a significant trauma and a total PCL-5 score of 33 or greater (Weathers et al., 2013b). Secondly, we also examined a measure of PTSD symptom severity. As the continuous summed PCL-5 score was heavily right-skewed (range 0–80; median 0; Skewness = 2.7; Kurtosis = 10.4), we derived an ordinal measure of PCL-5 score severity (score 0, including those not endorsing lifetime trauma on LEC (value 0); score 1–19 (value 1); score 20–39 (value 2); score 40–59 (value 3); score 60–80 (value 4)). Data were collected using the RedCap data collection tool (Harris et al., 2009, 2019).

#### 2.2.3 Confounders

We adjusted for the following confounders in our analyses; sex, maternal history of severe depression (reported by mother during pregnancy, (yes/no)), maternal smoking during pregnancy (yes/no), maternal social class (categorical variable indexing classes I-V of the UK Registrar General's occupational coding), and genetic risk for neuroticism as indexed by a continuous, normally-distributed polygenic risk score derived using the Smith et al. GWAS (Smith et al., 2016) at a p-threshold of 0.5.

### 2.3 Statistics

STATA version 15 was used for data analysis. Pearson correlation was used to examine the correlation within (over time) and between temperament traits. Logistic regression was used to estimate unadjusted and adjusted odds ratios (OR) and 95% confidence intervals (95% CI) of trauma and PTSD per SD increase in temperament traits and between temperament clusters. Ordinal regression was used to assess the relationship between temperament traits and PTSD symptom severity.

To minimize the potential for reverse causation, whereby early-life exposure to trauma led to a subsequent change in temperament traits, we conducted a sensitivity analysis where we examined the association between temperament and trauma whilst omitting individuals exposed to any trauma prior to age 5 from the analyses.

We used structural equation modelling using the ‘gsem’ command in Stata to determine the extent to which trauma exposure mediated the relationship between temperament traits and PTSD/PTSD symptom severity; this analysis was also adjusted for confounders. To examine whether temperament traits modified the association between trauma and PTSD/PTSD severity, we included temperament × trauma interaction terms and report LRT p-values for interactions under multiplicative models.

## 3 Results

### 3.1 Sample

Our final sample consisted of 2017 participants with complete data for temperament, trauma and PTSD measures, as well as data on confounders (see Suppl. Fig. 1).

Compared to the rest of the ALSPAC cohort, participants in our sample had a higher maternal socioeconomic status, and lower prevalence of maternal smoking during pregnancy, maternal depression, and exposure to a trauma aged 0–5 years (see Suppl. Table 2).

Suppl. Table 3 shows the temperament traits stratified by confounders. Male sex was associated with higher Activity, whereas higher Approach and Threshold were associated with female sex.

Adjusting for confounders made minimal difference to any of the results and therefore primarily the adjusted results are present below (see Supplement for unadjusted estimates).

### 3.2 Association between temperament traits and trauma

1178 (58.4%) participants in our sample were exposed to trauma ages 0–17. There was evidence that SD increases in levels of Activity, Adaptability, Intensity, Mood and Threshold were associated with a small increase in the odds of exposure to trauma (ORs ranging from 1.12 to 1.23) between ages 0–17. Estimates were little changed after adjusting for confounders (Table 2). There was little evidence of association with the other traits examined. Odds of trauma exposure was increased for participants with Medium (OR_adjusted_ 1.49, 95% CI 1.21, 1.84, *p* < 0.001) and Difficult (OR_adjusted_ 1.47, 95% CI 1.18, 1.84, *p* = 0.001) temperament clusters compared to those with an Easy temperament (see Table 2).

**Table 2:** Adjusted^a^ odds ratios (OR) and 95% confidence intervals (95% CI) between childhood temperament traits and trauma, PTSD, and PTSD symptoms.

	Trauma			PTSD diagnosis			PTSD symptoms		
Temperament traits	OR	95% CI	P value	OR	95% CI	P value	OR	95% CI	P value
**Activity**	1.18	1.07, 1.29	<0.001	1.13	0.94, 1.37	0.198	1.07	0.98, 1.18	0.121
**Rhythmicity**	1.06	0.97, 1.16	0.215	0.95	0.78, 1.14	0.566	1.01	0.93, 1.11	0.792
**Approach**	1.03	0.94, 1.13	0.506	1.07	0.88, 1.30	0.489	0.99	0.90, 1.09	0.871
**Adaptability**	1.13	1.03, 1.24	0.010	1.05	0.86, 1.27	0.645	1.07	0.97, 1.17	0.169
**Intensity**	1.23	1.12, 1.34	<0.001	1.27	1.05, 1.54	0.012	1.10	1.01, 1.21	0.034
**Mood**	1.12	1.02, 1.23	0.015	1.04	0.85, 1.27	0.685	1.03	0.93, 1.13	0.607
**Persistence**	1.04	0.95, 1.13	0.432	1.04	0.86, 1.26	0.675	0.98	0.89, 1.08	0.693
**Distractibility**	1.07	0.98, 1.17	0.140	0.94	0.78, 1.15	0.559	1.03	0.94, 1.13	0.532
**Threshold**	1.10	1.01, 1.20	0.037	1.06	0.88, 1.28	0.538	1.15	1.05, 1.26	0.003
**Temperament cluster**^b^									
**Medium**	1.49	1.21, 1.84	<0.001	1.15	0.74, 1.80	0.536	1.14	0.92, 1.42	0.225
**Difficult**	1.47	1.18, 1.84	0.001	0.96	0.59, 1.58	0.883	1.17	0.93, 1.47	0.183

a Adjusted for sex, maternal history of severe depression, maternal smoking during pregnancy, maternal socioeconomic status and genetic risk for neuroticism.

b Compared to baseline Easy temperament cluster.

In a sensitivity analysis, where we omitted individuals exposed to trauma early in childhood to minimize the likelihood that temperament traits were being influenced by exposure to trauma very early in childhood (reverse causation effects), we found that the associations with later childhood/adolescent trauma were almost unchanged for Activity and Intensity, and those for Adaptability, Mood and Threshold were weaker (see Suppl. Table 5). The association between a Medium temperament cluster and trauma was also relatively unchanged, while that for a Difficult cluster was weaker (Suppl. Table 5).

### 3.3 Association between temperament traits and PTSD

At age 23, 112 of the sample (5.5%, 95% CI 4.6%, 6.6%) met criteria for probable PTSD. Higher levels of Intensity were associated with a small increase in odds of having PTSD at age 23 (OR_adjusted_ 1.27, 95% CI 1.05, 1.54, *p* = 0.012). There was little evidence of association for any of the other traits examined (Table 2). There was little evidence that temperament clusters were associated with PTSD or PTSD symptom severity at age 23 years (Table 2).

When examining the outcome of PTSD symptom severity, we found evidence of associations with Intensity and Threshold but not with the other traits examined (Table 2).

### 3.4 Interaction analyses

Individuals exposed to trauma in childhood/adolescence had an approximately 2-fold increase in odds of having PTSD at age 23 (OR 1.92, 95% CI 1.26, 2.94, *p* = 0.002). There was some evidence that the association between trauma and PTSD diagnosis on a logistic scale was stronger in individuals with higher levels of trait Intensity (OR_adjusted_ 1.66, 95% CI 1.07, 2.55, *p* = 0.023), but this was not observed for PTSD symptoms (OR_adjusted_ 1.05, 95% CI 0.87, 1.28, *p* = 0.599). There was little evidence that the strength of association between childhood/adolescent trauma and adult PTSD differed according to any of the other temperament trait levels in early childhood (Table 3).

**Table 3:** Unadjusted and adjusted^a^ interaction odds ratios (OR) and 95% confidence intervals (95% CI) between temperament traits and trauma on PTSD and PTSD symptoms.

	PTSD diagnosis						PTSD symptoms					
	Unadjusted			Adjusted			Unadjusted			Adjusted		
Temperament traits	OR	95% CI	P value	OR	95% CI	P value	OR	95% CI	P value	OR	95% CI	P value
**Activity**	1.02	0.67, 1.55	0.934	1.02	0.67, 1.55	0.940	0.98	0.81, 1.18	0.835	0.98	0.81, 1.19	0.837
**Rhythmicity**	0.86	0.57, 1.31	0.482	0.89	0.58, 1.35	0.574	0.97	0.80, 1.17	0.717	0.98	0.81, 1.19	0.866
**Approach**	0.72	0.47, 1.10	0.130	0.70	0.46, 1.08	0.105	0.96	0.79, 1.16	0.649	0.94	0.78, 1.15	0.562
**Adaptability**	1.03	0.66, 1.58	0.907	1.02	0.66, 1.57	0.939	0.90	0.74, 1.10	0.314	0.90	0.74, 1.10	0.297
**Intensity**	1.65	1.07, 2.53	0.022	1.66	1.07, 2.55	0.023	1.06	0.87, 1.28	0.555	1.05	0.87, 1.28	0.599
**Mood**	1.03	0.66, 1.60	0.897	1.04	0.66, 1.62	0.878	1.05	0.86, 1.28	0.636	1.06	0.87, 1.29	0.588
**Persistence**	0.98	0.64, 1.50	0.934	1.01	0.66, 1.55	0.966	0.96	0.79, 1.17	0.700	0.98	0.81, 1.19	0.835
**Distractibility**	0.82	0.54, 1.27	0.378	0.81	0.53, 1.26	0.349	0.98	0.80, 1.19	0.813	0.97	0.80, 1.18	0.745
**Threshold**	0.92	0.61, 1.40	0.704	0.89	0.59, 1.36	0.601	0.97	0.80, 1.17	0.733	0.95	0.79, 1.16	0.628

a Adjusted for sex, maternal history of severe depression, maternal smoking during pregnancy, maternal socioeconomic status and genetic risk for neuroticism.

### 3.5 Mediation analyses

As Intensity was associated with both trauma and PTSD, we examined the extent to which the association between Intensity and adult PTSD was mediated by childhood/adolescent trauma (see Table 4). There was evidence that trauma mediated part of the relationship between Intensity and PTSD (Indirect OR_adjusted_ 1.08, 95% CI 1.01, 1.16, *p* = 0.024, proportion mediated 26.2%). Results were stronger when examining PTSD symptoms (Indirect OR = 1.08, 95% CI 1.04, 1.13, *p<*0.001; proportion mediated 50.4%).

**Table 4:** Unadjusted and adjusted^a^ odds ratios (OR) and 95% confidence intervals (95% CI) for mediation of the association between childhood Intensity and adult PTSD and PTSD symptoms by childhood/adolescent trauma.

	PTSD diagnosis			PTSD symptoms		
	OR	95% CI	P value	OR	95% CI	P value
**Indirect Effect**	1.08	1.01, 1.16	0.024	1.08	1.04, 1.13	<0.001
**Total Effect**	1.34	1.10, 1.64	0.003	1.17	1.06, 1.29	0.003
**% Mediated**	26.2%			50.4%		

a Adjusted for sex, maternal history of severe depression, maternal smoking during pregnancy, maternal socioeconomic status and genetic risk for neuroticism.

## 4 Discussion

### 4.1 Main findings

In this large population-based cohort study, we examined whether temperament traits in early childhood were associated with childhood/adolescent trauma and PTSD in early adulthood, and whether these traits moderated the relationship between trauma and PTSD. Our results show that traits relating to higher levels of intensity of reactions and greater activity, as well as more difficult temperament clusters were associated with subsequent exposure to trauma in the developmental period, and that these associations were not explained by socio-demographic, family history or genetic confounders. Our results also show that it is unlikely that these associations can be explained by reverse causation, whereby temperament traits in childhood result from exposure to trauma very early in childhood.

Of the traits and clusters examined, only Intensity was consistently associated with the development of PTSD at age 23 years, while Threshold trait levels were associated with more severe PTSD symptoms though not with PTSD onset. Trait Intensity modified the relationship between trauma and PTSD, suggesting that higher levels of trait Intensity are more likely to result in symptoms of PTSD after exposure to trauma. Trauma in childhood/adolescence mediated part of the association between trait Intensity and PTSD in adulthood. All effect sizes for the associations we observed were small.

### 4.2 Strengths and limitations

The main strength of our study is the use of a large prospective cohort with detailed measures. This means the study is well-powered, whilst recall bias and reverse causation are minimised.

However, the findings from our study should be interpreted in the context of the following limitations. First, although our findings were consistent after adjusting for what we considered to be the most important confounders (sex, family psychiatric history, social class and genetic risk for neuroticism), there may still be residual confounding if we omitted important confounders in our analyses, or if there is measurement error in those we adjust for, a limitation inherent in all observational designs. Second, whilst the potential for bias will have been reduced by the longitudinal nature of our data, it is nevertheless possible that individuals with particular traits are more likely to perceive events in their life as traumatic, or that they have increased recall of difficult experiences.

Third, as in any cohort study there was attrition of the sample over time, and participants with sufficient data to be included in our analyses differed from those omitted. As data were not missing completely at random, it is possible that our estimates are affected by selection bias, although both empirical and simulation data suggest that associations between childhood exposures and mental health outcomes in ALSPAC are unlikely to be strongly affected by selection bias (Wolke et al., 2009; Kounali et al., 2014).

Fourth, there was only weak to moderate correlation between the temperament measures at 6 and 24 months, suggesting that these traits are not highly stable over time. However, our results were similar when we examined the temperament traits separately at 6 and 24 months of age (results available on request). Fifth, the measure of PTSD used was a self-reported questionnaire rather than a clinical assessment, and only measured current rather than lifetime PTSD. This may have led to underestimating associations with PTSD and mediating effects of trauma in our study. Finally, when examining PTSD symptom severity as an outcome we made the assumption that those who did not endorse a lifetime traumatic event at that assessment (and therefore did not complete the PCL-5) scored 0 on the PCL-5. However, one-off measures usually underestimate lifetime prevalence, and this may have introduced measurement error in our estimates.

### 4.3 Possible mechanisms

Intensity of reaction and emotionality (a closely related concept) are key indicators of a difficult temperament (Rzsezutek et al., 2012) and have been shown to be associated with several different childhood and adolescent psychiatric disorders (Sayal et al., 2014; Bould et al., 2014). It is possible that children with increased Intensity are more at risk of trauma exposure as their increased reactivity (be it positive or negative) to what may seem like minor stimuli to others may cause frustration and irritation in care-givers and peers, leading to an increase in abuse and bullying.

Similarly, children who are more active and demanding might be more likely to elicit instances of physical or emotional abuse from a tired or frustrated caregiver, and the association we observed between higher Activity trait levels and subsequent trauma exposure is consistent with previous evidence that children with attention deficit hyperactivity disorder, where increased activity is a core feature, are at increased risk of abuse compared to other children (Tistarelli et al., 2020).

Furthermore, previous research has found that neuroticism, defined by a preponderance to anxious, negative moods and emotional instability, is the personality trait that is most strongly associated with PTSD (DiGangi et al., 2013; Cox et al., 2004; Mattson et al., 2018). This is thought to result from individuals with higher neuroticism having a lower threshold of response to stressful stimuli as they are more likely to perceive events in their environment as negative and distressing (Ehlers and Clarke, 2000, Stein et al., 2018), and might help explain our observation that the association between trauma and PTSD is stronger in individuals with higher levels of trait Intensity. However, interactions are often scale-dependant and usually difficult to replicate (Zammit et al., 2010), and therefore particular caution is needed when interpreting this result.

## 5 Implications of findings

Our research indicates that increased activity and reactivity in early childhood increase the likelihood of trauma exposure during the developmental period. As traumatic events increase the development of a variety of psychiatric disorders apart from PTSD, including depression, psychosis, conduct disorder and alcohol use disorders (Lewis et al., 2019), our findings suggest there might be a potential route to alter long-term outcomes of children with more extreme temperament traits if these traits can be modified, for example using psychosocial interventions. Replication of these findings in future studies could lead to greater awareness amongst clinicians of the potential adverse outcomes for children with more extreme temperament traits, and help inform prediction models for PTSD.

## 6 Conclusion

Specific temperament traits in early childhood are associated with an increased risk of developmental trauma. Stronger intensity of reaction to novel stimuli, and to a lesser extent threshold of responsiveness, were also associated with risk of PTSD in early adulthood. Childhood temperament traits offer a potential target for supportive interventions to reduce adverse mental health outcomes in young people.

## Author statement

Chantelle Wiseman: Data Curation, Formal Analysis, Visualisation, Writing- Original draft preparation. Jazz Croft: Methodology, Data Curation, Writing- Review and Editing. Stan Zammit: Conceptualization, Supervision, Methodology, Writing- Review and Editing.

## Funding

The UK Medical Research Council and Wellcome Trust (Grant ref: 217065/Z/19/Z) and the 10.13039/501100000883University of Bristol provide core support for ALSPAC. This publication is the work of the authors and Chantelle Wiseman and Stan Zammit will serve as guarantors for the contents of this paper.

A comprehensive list of grants funding is available on the ALSPAC website (http://www.bristol.ac.uk/alspac/external/documents/grant-acknowledgements.pdf); This research was specifically funded by 10.13039/501100000265the Medical Research Council (MRC) grant number MR/M006727/1. GWAS data was generated by Sample Logistics and Genotyping Facilities at Wellcome Sanger Institute and LabCorp (Laboratory Corporation of America) using support from 23andMe.

This study was supported by an Institutional Strategic Support Fund round 3 (ISSF3) grant from 10.13039/501100000866Cardiff University, The Wellcome Trust GW4 CAT scheme (grant number 216280/Z/19/Z), and 10.13039/501100000265the Medical Research Council (MRC) grant MR/M006727/1. SZ is supported by the NIHR Biomedical Research Centre at University Hospitals Bristol NHS Foundation Trust and the 10.13039/501100000883University of Bristol. The views expressed in this publication are those of the authors and not necessarily those of the NHS, the National Institute for Health Research or the Department of Health and Social Care.

## Presentation

An abstract of an earlier version of this work was presented as a poster and short talk at the 2019 World Psychiatric Association Congress of Psychiatry.

## Ethics approval

Informed consent for the use of data collected via questionnaires and clinics was obtained from participants following the recommendations of the ALSPAC Ethics and Law Committee at the time.

## Consent to participate

All participants consented to be involved in the ALSPAC study.

## Consent to publication

All participants consented to be involved in the ALSPAC study.

## Availability of data and materials

The ALSPAC database is available to researchers on request.

## Code availability

We are happy to make our codes available to researchers on request.

## Declaration of competing interest

The authors assert they have no conflict of interest with any organisation or entity with any financial interest or non-financial interest in the subject matter or materials discussed in this manuscript.

## Unlisted references

Ehlers et al., 2000; Stein et al., 2018;.
