# Supplementary material for: Examining the relationship between early childhood temperament, trauma, and post-traumatic stress disorder
Source: J Psychiatr Res. 2021 Dec;144:427–33. doi: 10.1016/j.jpsychires.2021.10.004 (PMC8670596; doi:10.1016/j.jpsychires.2021.10.004)
Supplement: Multimedia component 1 [file mmc1.docx]

**Supplement**

Supplementary Figure 1: Numbers of participants providing data for different measures and numbers included in the different analyses.

Supplement Table 1: Correlation within temperament traits measured at 6 and 24 months of age

| **Temperament trait** | **Correlation (Pearson’s)** | **P value** |
| --- | --- | --- |
| **Activity** | 0.30 | <0.001 |
| **Rhythmicity** | 0.41 | <0.001 |
| **Approach** | 0.25 | <0.001 |
| **Adaptability** | 0.24 | <0.001 |
| **Intensity** | 0.20 | <0.001 |
| **Mood** | 0.38 | <0.001 |
| **Persistence** | 0.34 | <0.001 |
| **Distractibility** | -0.05 | 0.020 |
| **Threshold** | 0.27 | <0.001 |

Supplementary Table 2: Differences in socio-demographic characteristics and childhood trauma between our sample and the rest of the ALSPAC cohort

|  | **Missing from final sample** | **Final sample** | **P value** |
| --- | --- | --- | --- |
|  | **N (%)** | **N (%)** |  |
| **Female sex** | 6093 (46.8%) | 1255 (62.2%) | <0.001 |
| **High maternal social class^a^** | 6336 (78.3%) | 1764 (87.5%) | <0.001 |
| **Maternal history of severe depression** | 1021 (9.7%) | 115 (5.7%) | <0.001 |
| **Maternal smoking in pregnancy** | 3046 (27.0%) | 260 (12.9%) | <0.001 |
| **Trauma age 0-5** | 2303 (27.3%) | 386 (19.4%) | <0.001 |

1. Social classes 1-3 non-manual (variable dichotomized for table)

Supplementary Table 3: Temperament traits (mean) stratified by confounders

|  | **Sex** | |  | **Family history^a^** | |  | **Higher social class^b^** | |  | **Maternal smoking** | |  | **Neuroticism genetic risk^c^** | |  |
| --- | --- | --- | --- | --- | --- | --- | --- | --- | --- | --- | --- | --- | --- | --- | --- |
|  | **Male** | **Female** | **p** | **Yes** | **No** | **p** | **Yes** | **No** | **p** | **Yes** | **No** | **p** | **Higher** | **Lower** | **p** |
| **Activity** | 0.10 | -0.07 | <0.001 | 0.07 | -0.01 | 0.404 | 0.00 | -0.05 | 0.500 | 0.11 | -0.03 | 0.442 | -0.07 | 0.01 | 0.655 |
| **Rhythmicity** | -0.07 | -0.02 | 0.312 | 0.07 | -0.04 | 0.262 | -0.05 | 0.07 | 0.078 | -0.21 | -0.01 | 0.516 | -0.06 | -0.03 | 0.139 |
| **Approach** | -0.16 | 0.05 | <0.001 | -0.04 | -0.03 | 0.926 | -0.04 | 0.07 | 0.105 | -0.15 | -0.01 | 0.155 | -0.01 | -0.03 | 0.606 |
| **Adaptability** | -0.08 | -0.12 | 0.375 | -0.05 | -0.10 | 0.556 | -0.11 | -0.01 | 0.133 | -0.10 | -0.10 | 0.954 | -0.05 | -0.11 | 0.237 |
| **Intensity** | -0.01 | -0.01 | 0.983 | 0.14 | -0.02 | 0.104 | -0.02 | 0.04 | 0.357 | 0.20 | -0.05 | 0.009 | 0.04 | -0.03 | 0.531 |
| **Mood** | 0.02 | -0.02 | 0.354 | 0.07 | -0.01 | 0.365 | -0.01 | 0.02 | 0.694 | -0.11 | 0.01 | 0.235 | 0.01 | -0.01 | 0.382 |
| **Persistence** | 0.01 | -0.07 | 0.104 | -0.11 | -0.03 | 0.411 | -0.03 | -0.09 | 0.352 | -0.16 | -0.02 | 0.002 | -0.05 | -0.04 | 0.952 |
| **Distractibility** | 0.02 | 0.02 | 0.938 | -0.04 | 0.02 | 0.491 | 0.02 | -0.01 | 0.607 | -0.13 | 0.04 | 0.001 | -0.04 | 0.03 | 0.315 |
| **Threshold** | -0.17 | 0.08 | <0.001 | 0.08 | -0.02 | 0.326 | -0.03 | 0.07 | 0.146 | 0.11 | -0.03 | 0.019 | -0.01 | -0.02 | 0.816 |

Footnote: a. Family history of severe maternal depression measured during pregnancy; b. Social class 1-3 non-manual (variable dichotomized for table); c. Polygenic risk score (dichotomised at 80th percentile for table)

Supplementary Table 4: Unadjusted odds ratios (OR) and 95% confidence intervals (95% CI) between childhood temperament traits and trauma, PTSD diagnosis and PTSD symptoms

|  | **Trauma** | | | **PTSD diagnosis** | | | **PTSD symptoms** | | |
| --- | --- | --- | --- | --- | --- | --- | --- | --- | --- |
| **Temperament traits** | **OR** | **95% CI** | **P value** | **OR** | **95% CI** | **P value** | **OR** | **95% CI** | **P value** |
| **Activity** | 1.19 | 1.08, 1.30 | <0.001 | 1.11 | 0.92, 1.34 | 0.296 | 1.06 | 0.97, 1.16 | 0.219 |
| **Rhythmicity** | 1.06 | 0.97, 1.15 | 0.205 | 0.96 | 0.80, 1.16 | 0.668 | 1.02 | 0.93, 1.11 | 0.702 |
| **Approach** | 1.01 | 0.93, 1.11 | 0.758 | 1.10 | 0.91, 1.33 | 0.340 | 1.01 | 0.92, 1.11 | 0.833 |
| **Adaptability** | 1.13 | 1.04, 1.24 | 0.006 | 1.05 | 0.86, 1.27 | 0.642 | 1.07 | 0.97, 1.17 | 0.171 |
| **Intensity** | 1.24 | 1.14, 1.36 | <0.001 | 1.28 | 1.07, 1.55 | 0.008 | 1.12 | 1.02, 1.22 | 0.018 |
| **Mood** | 1.12 | 1.02, 1.23 | 0.014 | 1.03 | 0.84, 1.25 | 0.779 | 1.02 | 0.93, 1.12 | 0.715 |
| **Persistence** | 1.03 | 0.94, 1.12 | 0.559 | 1.01 | 0.83, 1.22 | 0.944 | 0.96 | 0.88, 1.05 | 0.384 |
| **Distractibility** | 1.05 | 0.98, 1.17 | 0.262 | 0.92 | 0.76, 1.12 | 0.412 | 1.02 | 0.93, 1.12 | 0.703 |
| **Threshold** | 1.09 | 1.00, 1.19 | 0.051 | 1.11 | 0.92, 134 | 0.266 | 1.18 | 1.08, 1.29 | <0.001 |
| **Temperament cluster comparator^a^** |  |  |  |  |  |  |  |  |  |
| **Medium** | 1.50 | 1.21, 1.84 | <0.001 | 1.17 | 0.75, 1.82 | 0.493 | 1.14 | 0.92, 1.42 | 0.235 |
| **Difficult** | 1.47 | 1.18, 1.84 | 0.001 | 1.00 | 0.61, 1.64 | 0.998 | 1.19 | 0.95, 1.50 | 0.133 |

1. Compared to baseline Easy temperament cluster

Supplementary Table 5: Unadjusted and adjusted^a^ odds ratios (OR) and 95% confidence intervals (95% CI) between childhood temperament traits and trauma, individuals who experienced trauma below the age of 5 excluded from sample

|  |  | | **Unadjusted** | | | | **Adjusted** | | |
| --- | --- | --- | --- | --- | --- | --- | --- | --- | --- |
| **Temperament traits** | **OR** | **95% CI** | | **P value** | **OR** | **95% CI** | | **P value** |  |
| **Activity** | 1.21 | 1.10, 1.33 | | <0.001 | 1.20 | 1.09, 1.33 | | <0.001 |  |
| **Rhythmicity** | 1.04 | 0.95, 1.15 | | 0.386 | 1.04 | 0.95, 1.15 | | 0.387 |  |
| **Approach** | 0.96 | 0.87, 1.07 | | 0.472 | 0.99 | 0.89, 1.09 | | 0.779 |  |
| **Adaptability** | 1.09 | 0.99, 1.21 | | 0.093 | 1.09 | 0.99, 1.21 | | 0.090 |  |
| **Intensity** | 1.22 | 1.10, 1.34 | | <0.001 | 1.21 | 1.10, 1.34 | | <0.001 |  |
| **Mood** | 1.08 | 0.98, 1.20 | | 0.129 | 1.09 | 0.99, 1.21 | | 0.093 |  |
| **Persistence** | 1.03 | 0.93, 1.13 | | 0.588 | 1.04 | 0.94, 1.15 | | 0.466 |  |
| **Distractibility** | 1.03 | 0.93, 1.14 | | 0.561 | 1.04 | 0.94, 1.15 | | 0.418 |  |
| **Threshold** | 1.07 | 0.97, 1.18 | | 0.159 | 1.09 | 0.98, 1.20 | | 0.097 |  |
| **Temperament cluster^b^** |  |  | |  |  |  | |  |  |
| **Medium** | 1.49 | 1.19, 1.88 | | 0.001 | 1.48 | 1.18, 1.86 | | 0.001 |  |
| **Difficult** | 1.22 | 0.95, 1.56 | | 0.128 | 1.23 | 0.96, 1.59 | | 0.105 |  |

1. Adjusted for sex, maternal history of severe depression, maternal smoking during pregnancy, maternal socioeconomic status and genetic risk for neuroticism
2. Compared to baseline Easy temperament cluster
